# Draft Genome Sequence of *Lactobacillus plantarum* TL2766, a Strain with the Ability To Ferment Wakame

**DOI:** 10.1128/genomeA.01328-16

**Published:** 2016-11-23

**Authors:** Julio Villena, Lucila Saavedra, Elvira Maria Hebert, Yuki Masumizu, Nana Sato, A. K. M. Humayun Kober, Leonardo Albarracin, Wakako Ikeda-Ohtsubo, Seiya Makino, Katsunori Kimura, Sou Ohkawara, Haruki Kitazawa

**Affiliations:** a Reference Centre for Lactobacilli (CERELA-CONICET), Tucumán, Argentina; b Food and Feed Immunology Group, Tohoku University, Sendai, Japan; c Livestock Immunology Unit, International Education and Research Center for Food Agricultural Immunology (CFAI), Tohoku University, Sendai, Japan; d Food Science Research Laboratories, R&D Division, Meiji Co. Ltd., Kanagawa, Japan; e Agricultural & Veterinary Division, Meiji Seika Pharma Co., Ltd., Tokyo, Japan.

## Abstract

The genome sequence of *Lactobacillus plantarum* TL2766, a strain with the ability to ferment wakame (*Undaria pinnatifida*), is described here. The reads were assembled into contigs, with a total size of 3,310,195 bp. The genome information will be useful for further specific genetic studies of this strain and for its biotechnological applications.

## GENOME ANNOUNCEMENT

*Lactobacillus plantarum* TL2766 is a strain originally isolated from human adult feces that is able to grow and ferment wakame (*Undaria pinnatifida*), a seaweed often served in soups and salads in Japan. The TL2766 strain was evaluated *in vitro* and *in vivo* studies, and it was demonstrated that this bacterium does not have any detrimental effect on epithelial cells (1, 2) or antigen-presenting cells (3, 4) from porcine intestine origin. Moreover, *L. plantarum* TL2766 is able to survive the porcine gastrointestinal tract without any side effects (5). Those studies suggest that this strain is an interesting candidate for the development of new wakame-based food and feeds.

Here, we present a draft genome sequence of *L. plantarum* TL2766 that was sequenced using a whole-genome shotgun (WGS) strategy on an Illumina MiSeq sequencer. In total 30,037,481 paired reads with lengths of 300 bp were obtained, corresponding to a 2,722-fold coverage. Quality-filtered reads were assembled using the NGen (DNAStar) assembler, giving 34 contigs. The longest contig was 691,392 bp and the shortest 1,538 bp. Functional annotation of predicted genes was achieved using the RAST server and the NCBI Prokaryotic Genome Annotation Pipeline (PGAP) (6). tRNAs and rRNAs were identified by tRNAscan-SE and RNAmmer, respectively (7, 8).

The draft genome of *L. plantarum* TL2766 consists of 3,310,195 bp, with a mean G+C content of 44.4%. A total of 3,146 coding sequences (CDSs), 68 structural tRNAs, and nine rRNAs were predicted. Among all CDSs, 2,221 (71%) were assigned to known protein functions, while 925 (29%) CDSs remained as hypothetical proteins. Additionally, there are 341 RAST subsystems represented in the genome, which represent only 43% of the assigned sequences.

*In silico* studies revealed that TL2766 genome contains four genes encoding mucus-binding proteins (MBP) with 100% identity to MBP genes found in other *L. plantarum* strains. In addition, genes encoding homologues of bacteriocin ABC transporters, immunity proteins, as well as a structural gene of the plantaricin A bacteriocin were found in the TL2766 genome, suggesting the potential of this strain to produce antimicrobial compounds. Similar to other strains of the species, *L. plantarum* TL2677 contains genes responsible for exopolysaccharide biosynthesis. Gene clusters for the biosynthesis of biotin, thiamine, pyridoxine, and folate were also detected in the TL2766 genome.

The draft genome sequence of *L. plantarum* TL2766 will be useful for further studies of specific genetic features of this strain and for its biotechnological application in the development of novel food and feeds using wakame.

### Accession number(s).

This whole-genome shotgun project has been deposited at DDBJ/EMBL/GenBank under the accession no. LZXZ00000000. The version described in this paper is version LZXZ00000000.1.
